# A Microwave Ring-Resonator Sensor for Non-Invasive Assessment of Meat Aging

**DOI:** 10.3390/s16010052

**Published:** 2016-01-20

**Authors:** Muhammad Taha Jilani, Wong Peng Wen, Lee Yen Cheong, Muhammad Zaka ur Rehman

**Affiliations:** 1 Department of Electrical and Electronics Engineering, University Technology PETRONAS, Bandar Seri Iskandar 32610, Malaysia; mtaha.jilani@gmail.com (M.T.J.); zakarahman@gmail.com (M.Z.R.); 2 Department of Fundamental and Applied Science, University Technology PETRONAS, Bandar Seri Iskandar 32610, Malaysia; lee_yencheong@petronas.com.my

**Keywords:** dielectric properties, complex permittivity, dielectric spectroscopy, meat quality, microwave sensor, microstrip ring-resonator, biosensor

## Abstract

The assessment of moisture loss from meat during the aging period is a critical issue for the meat industry. In this article, a non-invasive microwave ring-resonator sensor is presented to evaluate the moisture content, or more precisely water holding capacity (WHC) of broiler meat over a four-week period. The developed sensor has shown significant changes in its resonance frequency and return loss due to reduction in WHC in the studied duration. The obtained results are also confirmed by physical measurements. Further, these results are evaluated using the Fricke model, which provides a good fit for electric circuit components in biological tissue. Significant changes were observed in membrane integrity, where the corresponding capacitance decreases 30% in the early aging (0D-7D) period. Similarly, the losses associated with intracellular and extracellular fluids exhibit changed up to 42% and 53%, respectively. Ultimately, empirical polynomial models are developed to predict the electrical component values for a better understanding of aging effects. The measured and calculated values are found to be in good agreement.

## 1. Introduction

**Figure 1:**
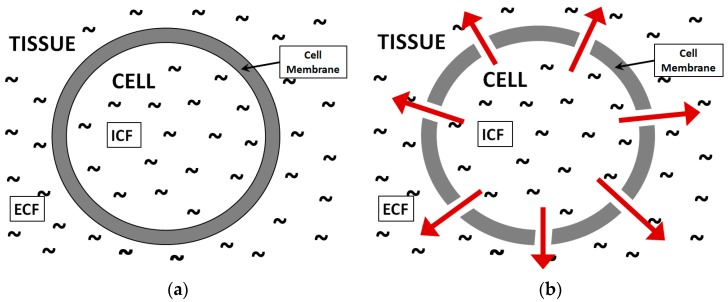
Fluids within a muscle, intracellular (ICF) and extracellular (ECF) compartment fluids. (**a**) Fresh muscle with intact cell membrane; (**b**) muscle with permeable cell membrane.

The electrical characteristics of food materials have been analyzed for many years to determine their physical, chemical and physiological states. Particularly for meat products, the non-invasive and online assessment of these properties enables the industry to evaluate meat quality effectively. Meat quality is usually evaluated based on physical attributes including pH, color, water holding capacity (WHC) and cook loss [1]. The water holding capacity (WHC) is considered the most important attribute in meat quality, since water is the main constituent, holding about 75% of the total body mass. As a matter of fact, the strong correlation between moisture content and dielectric permittivity allows researchers to evaluate the state of meat usinga microwave technique [2]. Several research studies have reported the determination of these attributes through examination of dielectric properties [3,4,5,6]. Discrimination of low quality classes of meat, such as pale, soft and exudative (PSE) and dark, firm and dry (DFD) is carried out at 500 MHz to 20 GHz frequency using an open-ended coaxial probe [7]. In the early period of animal slaughter, the muscle glycolysis procedure triggers rigor mortis (stiffness) in the animal carcass, where the pH level approaches the myofibrillar protein isoelectric point (pH near to 5.4) which results in high ionic mobility in muscle fluids [8]. This ultimately increases the ionic conductivity, and apparently changes the dielectric properties significantly. Results obtained from [7] suggest that determination between PSE and DFD grade meat is possible in early postmortem period, and the changes in water holding capacity (WHC) are more viable at two punctual frequencies (500 MHz and 10 GHz). Similarly, structural changes produced during the aging have been identified in the 200 MHz to 20 GHz frequency range [9]. It is observed that during the aging period, the dielectric constant decreases at all frequencies while the loss-factor increases at frequencies below 4 GHz and beyond that it remains constant. This effect on dielectric properties is mainly contributed by the water loss during storage [9]. The build-up of lactic acid during the aging period is known to be a main reason for reduction in water holding capacity (WHC). Therefore, different concentrations of lactic acid solution are evaluated at microwave frequencies ranging from 500 MHz to 20 GHz [10]. Good correlations are found among lactic acid, ATP and IMP content with the loss factor at 500 MHz, 915 MHz and 1 GHz frequencies. The aging period induces structural change, particularly in membrane integrity, insulating properties of cellular membrane, and intracellular fluid (ICF) and extracellular fluid (ECF) composition [11]. These structural changes can also be produced by freezing. The permeability of cell membranes produces a mix of extracellular and intracellular fluids, and eventually loss of moisture (as can be seen in Figure 1). This increases the conductivity of muscle and therefore produces substantial changes in dielectric properties.

The ability to measure the dielectric properties of meat, and to correlate them with the amount of moisture content, offers new opportunities to identify moisture lost during the aging period. In order to determine the moisture lost, several microwave methods are being reported. Among them, the coaxial dielectric probe method is widely used to evaluate permittivity measurements. It has high accuracy but is still subject to errors. The main source of error includes radiation effects [12], the air-gap problem and cable movement during measurements [13]. Further, while evaluating the physical properties it would be useful to measure the average permittivity of a material. Considering these aspects, recently, a highly sensitive microwave resonator sensor has been proposed to evaluate the moisture amount [14]. The ring resonator is based on the resonance mechanism, which offers not just high accuracy but also simple, cost-effective and fast measurements [15]. In this method sample loading and unloading is easier, thus enabling the evaluation of large and irregularly shaped samples. The ring-resonator sensor has shown commendable accuracy compared to the well-established coaxial probe technique [16]. In addition to this, the ring resonator technique is also evident in other biological applications, such as for hyperthermia [17] and liquid solvent sensing [18].

In this study a microwave ring-resonator sensor has been used for non-invasive assessment of meat aging. For this purpose, broiler meat is investigated, since it is considered the second highest source of meat after pork, globally. The physical and the electrical measurements are carried out over a four week period, and their results are investigated to assess the moisture lost during the aging period. These results are then used to analyze the impact of moisture lost on the electrical properties of the muscle. Further, the obtained data is evaluated using the Fricke model, which provides a good fit for electric parameters. This will help to identify the relationship between electrical and physical characteristics.

## 2. Microwave Ring-Resonator Sensor: Design and Theoretical Analysis

### 2.1. Design and Development

**Figure 2:**
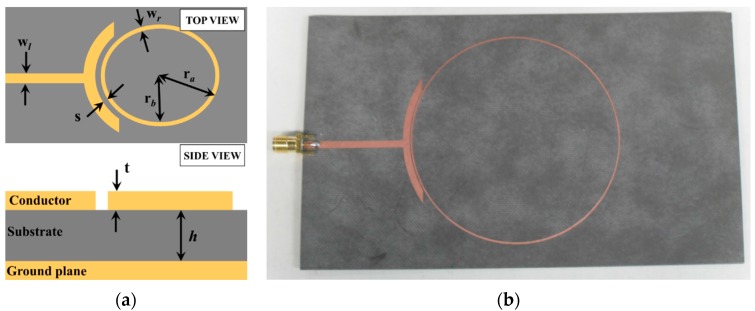
Highly sensitive microwave ring-resonator sensor. (**a**) Illustration; (**b**) Fabricated prototype.

**Figure 3:**
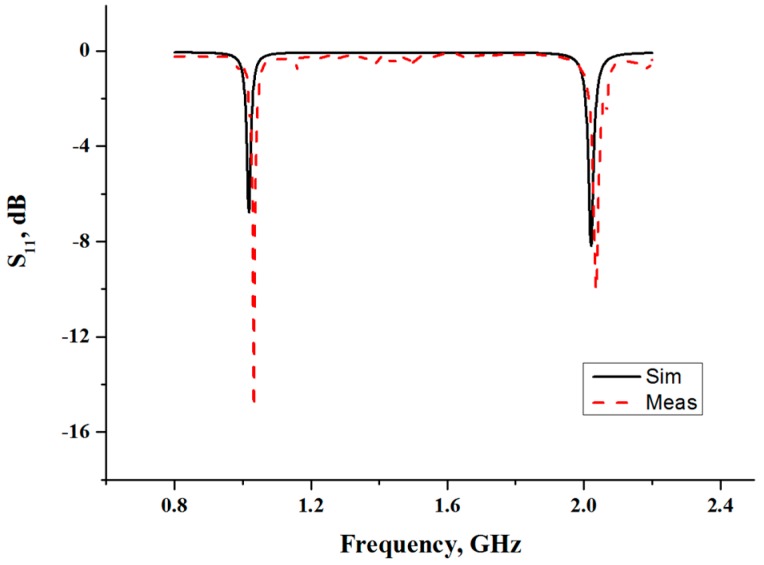
Simulated and measured resonance frequency response of a microwave ring-resonator sensor (without sample).

A single-port microstrip ring-resonator with a resonant frequency of 1 GHz is designed on Ansys HFSS software. The Roger RT/Duriod 5880 substrate with its dielectric constant of 2.2 and a height of 787 μm is used for the sensor structure, as reported in [14]. All design parameters of the microstrip ring-resonator sensor are tabulated in Table 1. The sensor (Figure 2) is then fabricated by using the standard lithography process. The frequency responses from simulation and measurement are then compared, over a frequency ranging from 200 MHz to 10 GHz. However, only the fundamental frequency response is considered for further evaluation, due to its greater sensitivity than harmonic frequencies. Simulation and measurement results over the frequency range 800 MHz to 2.3 GHz are presented in Figure 3. The resonator shows periodic resonances over the regular interval of 1 GHz frequency, in the absence of any overlay sample. The simulated and measured responses of the resonance frequency are in good agreement; however, some discrepancy is observed for the return-loss level. This is mainly due to limitations on accuracy, which are introduced during the fabrication process. Furthermore, the surrounding environment (such as temperature or humidity) can also affect losses.

**Table 1:** Design parameters of a highly sensitive microwave ring-resonator sensor.

Design Parameter	Value
Substrate dielectric constant (*ɛ_r_*)	2.2
Dissipation factor (*tan* *δ*)	0.0009
Ring outer-radius (*r_a_*)	34.87 mm
Ring inner-radius (*r_b_*)	34.27 mm
Feed width (*w_l_*)	35 mm
Ring width (*w_r_*)	2.4 mm
Coupling gap (*s*)	600 µm
Conductor strip (*t*)	17.5 µm
Substrate height (*h*)	787 µm

### 2.2. Theoretical Analysis

**Figure 4:**
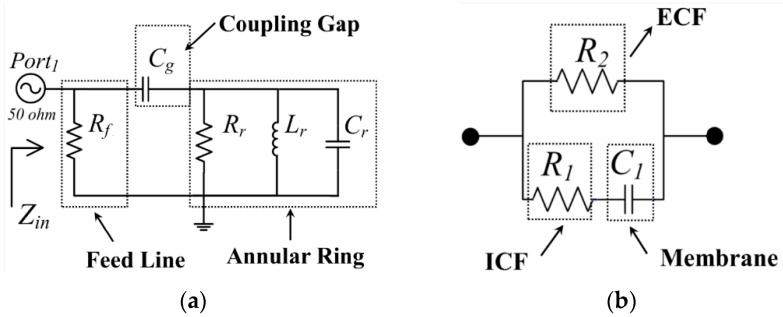
Equivalent RLC circuit of (**a**) Capacitively coupled ring resonator; (**b**) Fricke model representing the biological tissue.

The most straightforward way to analyze the ring structure is an equivalent RLC circuit. As shown in Figure 4a, the ring-resonator can be presented as a simple parallel RLC circuit [19]. The equivalent circuit consists of the ring’s capacitance (*C_r_*), inductance (*L_r_*) and resistance (*R_r_*), and also includes the capacitive coupling (*C_g_*) and feed line losses (*R_f_*). To determine these equivalent lumped components the standard transmission line theory can be used, where the ring RLC elements are described as [20].

(1) $$R_{r}=\frac{1}{\left(\frac{1}{R_{rad}}+\frac{1}{R_{cond}}+\frac{1}{R_{dielec}}\right)}(\Omega )$$

(2) $$C_{r}=\frac{\pi }{Z_{0}\varpi_{0}}\;(\text{F})$$

and

(3) $$L_{r}=1/\varpi_{0}^{2}C_{r}\;(\text{H})$$

where *R_rad_*, *R_cond_* and *R_dielec_* are the losses associated with radiation, conductor and dielectric material, as reported elsewhere [21]. Further, *Z_0_* and *ω_0_* are the characteristic impedance and angular frequency, respectively. The coupling-gap capacitance (*C_g_*), which coupled the power to the resonator through feed lines, is extensively analyzed [22]. The closed-form expression to cater its effect is defined by

(4) $$C_{g}=\frac{\left(C_{odd}-\frac{C_{even}}{2}\right)}{2}$$

(5) $$\begin{array}{l} \frac{C_{odd}}{w}={\left(\frac{s}{w}\right)}^{m_{0}}e^{k_{0}}\;\text{pF}/\text{m}\\ \frac{C_{even}}{w}=12{\left(\frac{s}{w}\right)}^{m_{e}}e^{k_{e}}\;\text{pF}/\text{m} \end{array}$$

where *s* is the gap size, *w* is the strip width, *m_e_* and *m_o_* are the even and odd mode values, and *k_e_* and *k_e_* are the even and odd constants [22]. The losses occurring due to the feed line (*R_f_*) can be obtained by using a computer program to fit its value to simulation data.

Figure 4b depicts a Fricke model [2], which provides a good fit to electric circuit components in the biological tissue. The tissue contains extracellular fluid (ECF), which behaves as an electrolyte which mainly comprises Na^+^ and Cl^−^ ions [11]. The resistance *R_2_* represents the losses associated with the ECF. Similarly, the resistance *R_1_* represents the associated losses produced by another fluid, *i.e.*, intra-cellular fluid (ICF). The cellular membrane, which has insulating properties, behaves likes a capacitor between two electrolytes and is represented as *C_1_* capacitance.

The dielectric material loaded ring-resonator can be analyzed by considering the parallel connection between the tissue and ring circuits, as reported earlier [19]. Initially, the individual transfer matrix of each component of tissue is determined and their corresponding *Y*-parameters are obtained:

(6) $${\left[Y\right]}_{R1}=\left[\begin{array}{cc} \frac{1}{R_{2}} & -\frac{1}{R_{2}}\\ -\frac{1}{R_{2}} & \frac{1}{R_{2}} \end{array}\right]$$

(7) $${\left[Y\right]}_{C1}=\left[\begin{array}{cc} \frac{1}{\frac{-j}{\varpi C_{1}}+R_{1}} & -\frac{1}{\frac{-j}{\varpi C_{1}}+R_{1}}\\ -\frac{1}{\frac{-j}{\varpi C_{1}}+R_{1}} & \frac{1}{\frac{-j}{\varpi C_{1}}+R_{1}} \end{array}\right]$$

and

(8) $${\left[Y\right]}_{Cg}=\left[\begin{array}{cc} \frac{1}{\frac{-j}{\varpi C_{g}}} & -\frac{1}{\frac{-j}{\varpi C_{g}}}\\ -\frac{1}{\frac{-j}{\varpi C_{g}}} & \frac{1}{\frac{-j}{\varpi C_{g}}} \end{array}\right]$$

Adding [*Y*]_R1_ + [*Y*]_C1_ + [*Y*]_Cg_ will give the overall *Y*-parameter, which is then transform back into the ABCD parameter [19]. This ABCD matrix can be used to evaluate the input impedance (*Z_in_*) of the whole circuit, using:

(9) $$Z_{in}=\frac{AZ_{L}+B}{CZ_{L}+D}$$

Eventually, the return loss (S_11_) of the sample loaded ring-resonator sensor can be obtained by:

(10) $$S_{11}=\frac{Z_{in}-Z_{0}}{Z_{in}+Z_{0}}$$

## 3. Experimental

### 3.1. Sample Handling and Preparation

**Figure 5:**
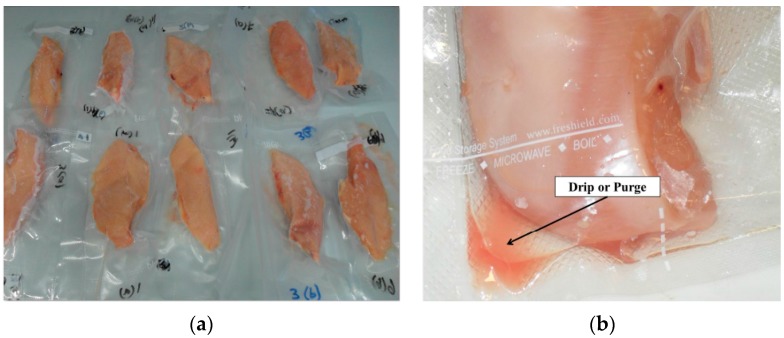
Studied broiler breast fillets. (**a**) Samples stored in freezer; (**b**) Equilibrated sample with collected drip.

Fresh broiler carcasses were collected from the local supplier at 45 min postmortem time and then immediately transported to the laboratory in an ice-filled box. Breast fillets (pectoralis major) from the right half of the carcass were excised and their weight was measured with weighing scale (Ohaus, Valor Precision 3000, New York, NY, USA). The left half of the breast remained intact with the carcass for the physical analysis. All samples were then vacuum packed by using a vacuum sealer (Fresheild, Elite III, Malaysia) and then stored at a chilling temperature at 4 °C (Figure 5a). Subsequent measurements were performed after 0, 1, 2, 7, 14, 21 and 28 days. On each measurement day, samples were taken out of the refrigerator and allowed to equilibrate at room temperature for 3 to 4 h, as reported elsewhere [9]. The cubic samples (30 × 35 × 15 mm, approximately) were obtained by trimming the fillet by using a sharp cutter. Special care was taken to extract samples of similar size, considering the material density influence on measurements [23]; however, due to its malleable nature a minor variation was found in size, which was negligible [24]. Since ageing can affect the anisotropy of muscles [25], dielectric measurements were taken both parallel and perpendicular to the fiber direction.

### 3.2. Physical Measurements—pH, Color and Temperature

The relationship between meat quality, muscle pH, and color is well established [26,27]. Therefore, to identify that relationship, mean values of pH and CIE (Color L*, a*, and b*) were determined. The pH measurements were taken with a portable pH meter (Luton, PH-220S, China) by inserting the electrode approximately 10 mm into a sample. Initially it was measured at 8 h and 24 h postmortem time, and then it was recorded for 2D, 3D, 7D, 14D, 21D and 28D periods. Over the whole aging period, the meter was calibrated every time using standard calibration liquids provided with the product. For the refrigerated samples, once they were equilibrated at room temperature, the pH measurements were carried out before deboning of samples.

Meat color (CIE L*, a*, and b* values) was measured on the dorsal surface (bone side) using a Chroma-meter (Konica Minolta, CR-300, Osaka, Japan) at 1.5 h postmortem time. Fresh broiler fillets were selected for the color measurements which were free from any obvious color defects, such as blood spots or bruises. The Chroma-meter was used to measure the reflectance between 400 and 700 nm, while the CIE values were calculated on a D65 illuminant, as reported elsewhere [24]. Prior to measurements white and black color tiles were used for the calibration. Three measurements were taken for each sample, and their average value was further considered. For the aging and refrigerated samples measurements, moisture at the surface of samples was gently removed using absorbent tissue prior to color analyses. Particularly in the aging study, once the samples were measured the same areas of the samples were marked, such that the same surfaces of the samples could be used for repeated measurements.

Muscle temperature has a significant impact on meat tenderness during aging and accelerates the rate of pH decline [27]. Therefore, temperature of fresh fillets was measured with a probe-thermometer (Testo, Mini Thermometer 560), starting at 1.5 h postmortem time with regular intervals of 30 min until 24 h postmortem time. The temperature probe was inserted into the sample and left until it showed a constant value for temperature. During the study, the temperature of each fillet was measured before deboning the sample for the measurements. This ensured the equilibrium state of sample temperature with room temperature.

### 3.3. Electrical Measurements

**Figure 6:**
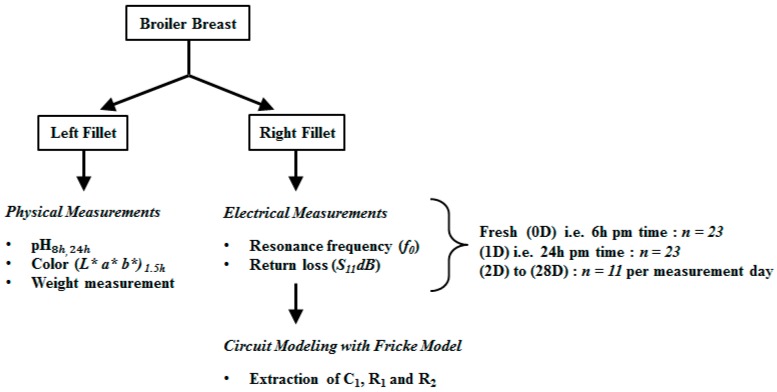
Experimental design for physical and electrical measurements.

The enhanced coupled high impedance microstrip-ring resonator was connected to an Agilent E8363C vector network analyzer and prior to measurements, it was calibrated with an open circuit, short circuit and load circuit using an Agilent standard calibration-kit. The VNA was configured to provide the S_11_ parameter for a range of 500 MHz to 20 GHz with 1980 frequency points. Initially, measurements were taken with unloaded resonator, once the unloaded resonance frequency was determined. The resonator was loaded with cubic samples (with a size of 30 × 35 × 15 mm, approximately), which were partially overlaid on the coupling region of the ring-resonator. Triplicate measurements were performed for the dielectric measurements of each sample. Samples were placed in a manner such that the orientation of fiber was perpendicular to the direction of electric field. Measurements were conducted at a controlled temperature of 25 ± 2 °C and humidity within the 55 RH% ± 5 range. The experimental design for both physical and electrical measurements is shown in Figure 6.

## 4. Results and Discussion

**Figure 7:**
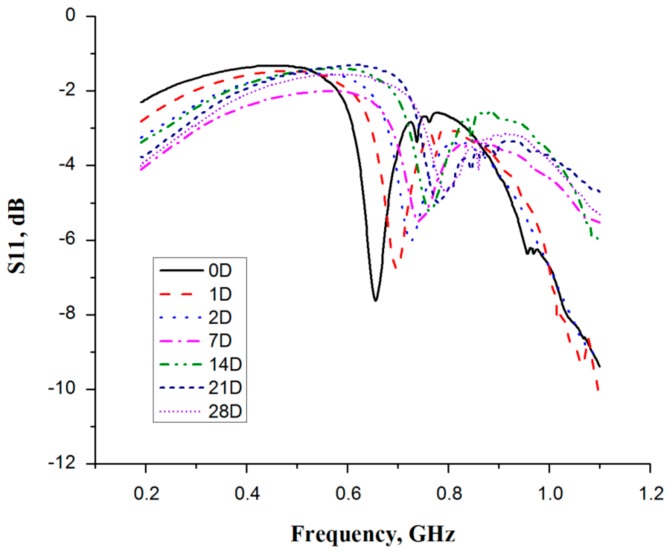
Resonance frequency observed for the meat stored up to four weeks (D = days).

The effect of meat aging was determined over four weeks post-mortem. The physical properties, including color and pH mean values, are presented in Table 2. The measured color (L*, a* and b*) values are close to those observed for the low quality meat, such as pale, soft and exudate (PSE) meat. Similarly, the pH values obtained at 8 h and 24 h postmortem time are related to those of low quality grade meat. The values are close to those published in the literature [28,29]. For the electrical measurements, overall seven observations were taken during the studied period. On each measurement day, the behavior of the resonance frequency as observed by using a microwave resonator sensor. Figure 7 shows the response of the resonance frequency for fresh (6 h after slaughter) to aged (28 days after slaughter) pectoralis major muscles from a broiler chicken (samples with similar physical characteristics are used for this purpose). It can be seen from the plot (Figure 7) that the fresh samples (0D) have the highest frequency shift of all the samples. However, over the storage period, the measured resonance frequency of the other samples is shifted toward the ring’s fundamental-frequency. This behavior is expected, since a reduction in the water holding capacity of the samples over the aging period causes an increase in drip-loss, thus reducing the amount of resonance shift.

**Table 2:** Measured physical properties of fresh broiler meat.

Physical Properties		Measured Value
Color ^1^	L*	57.61
	a*	1.77
	b*	5.19
pH ^2^	8 h	5.67
	24 h (postmortem time)	5.44

^1^ At 1.5 h postmortem time; ^2^ *n = 23* per mean with a variance of 0.0109, SD = 0.1044.

The main reason for this drip-loss is due to structural degradations, where the changes in intracellular and extracellular fluid composition took place [11,30]. The reduction in WHC changes the permittivity of meat samples, where the dielectric constant decreases continuously over the studied period. The change in the dielectric constant is significant for fresh (0D) sample, *i.e.*, 6 h postmortem time, but as the time progresses (0D to 28D) the amount of resonance shift is reduced. In this study, the shift observed with respect to unloaded resonance for the first day (0D *i.e.*, 6 h postmortem time) was 345 MHz, whereas in fourth week of aging (21D) this difference was reduced to 206 MHz. The change, or more particularly, amount of resonance shift was significant for the fresh (0D) sample measurement, while with passage of time, that is until 21D, the amount of resonance shift was reduced. These substantial changes in resonance frequency are mainly caused by the reduction in WHC during the storage period. It was determined that during the storage period the myofibrillar proteins (*i.e*., actin and myosin) undergo a progressive denaturation, which even involves the bond breaking [31]. Since most of the muscle water is present within the myofibrils, a general hypothesis for explaining drip loss is that it originates from shrinkage of myofibrils after death, causing the water to be expelled into the extracellular space [32]. The shrinkage of fiber expels the water from intracellular spaces to the extracellular spaces, and from where this water ultimately exudates in the form of drip. Since, myosin makes up approximately 50% of myofibrillar proteins, it causes a considerable amount of purge-loss [33]. Additionally, with conventional freezing the formation of large ice-crystals causes expansion and even rupture of the cell membrane [34]. Upon thawing of such muscles, the water is purged from the damaged cellular structure. This also increases the drip-loss in addition to the low pH level.

Other than change in resonance frequency, it is observed that the increase in bandwidth of the notches was also apparent in the obtained results. As described earlier, the loss-factor of the samples increases with increasing storage time, whereas this increase causes noticeable changes in the return-loss and the bandwidth of the resonance. However, as time progresses the resolution of this change in amplitude and bandwidth decreases. The increase of loss factor with storage time is due to the increase in ionic liquid volume. The intracellular fluid (ICF) mixes into extracellular fluids (ECF), increases the amount of ionic liquid, and apparently, increases conductivity. The abundant availability of free ions in these electrolytes provides transportation for electric charges [4]. As described earlier, the main contributing ions to support the current passage are Na^+^, K^+^ and Cl^−^, where the release of these ions is not only pH-dependent but also due to structural degradation during the storage period [35]. In addition Mg^2+^ and Ca^2+^ strongly bind to myosin proteins; however, when the pH reached the isoelectric point, the cancellation of charges releases these two cations.

Anisotropy of the meat sample was also analyzed; the measurements were taken in parallel and perpendicular to the fiber directions at 6 h and 1D postmortem time. The change in resonance frequency due to anisotropic variation is minimal, in both measurement periods. Therefore, further study on meat anisotropy was not conducted. However, due to reater dielectric sensitivity of samples with direction placement perpendicular to fiber [35], in further studies perpendicular orientation was used for the measurements.

**Figure 8:**
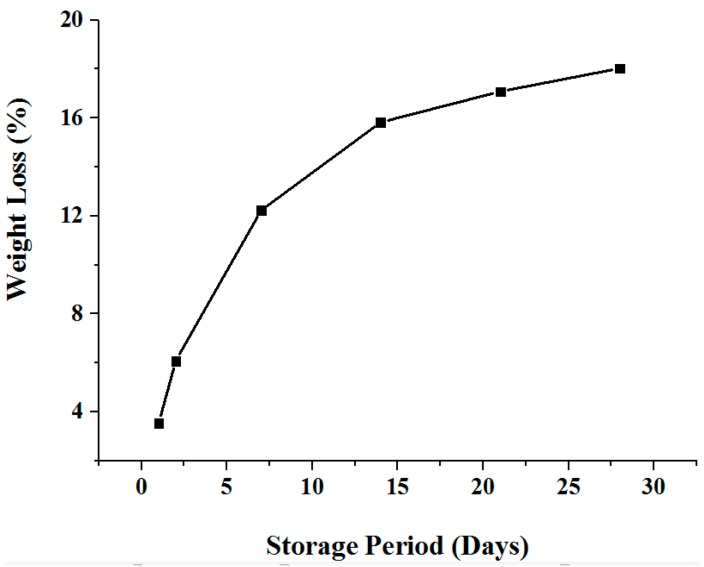
Weight loss of samples during storage period with respect to their fresh (0D) weight.

The loss of weight (due to reduction in WHC) of testing samples as determined by the difference between weight measured on 0D and weight on a given day, divided by the 0D weight. It can be seen from Figure 8 that over the whole storage period, weight loss increased with the storage days. This weight loss is due to an increase in drip-loss (for instance, drip-loss of a particular sample is shown in Figure 5b) and more specifically, reduction in water holding capacity.

### Equivalent Circuit Modeling for Storage Effect

**Figure 9:**
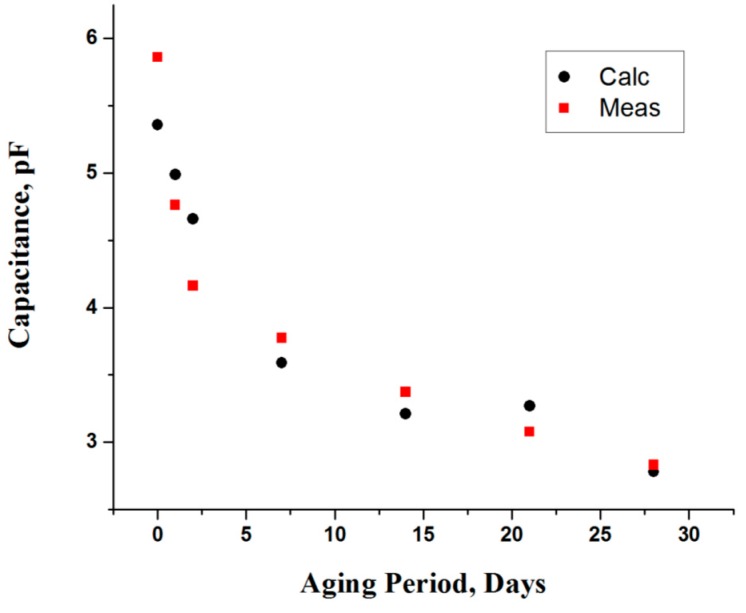
Capacitance (C_1_) of cell membrane as a function of aging period.

Meat equivalent circuit parameters are calculated from fittings of the resonance frequency and return loss plots to the Fricke model. The cellular membrane, which is characterized as capacitance (C_1_), deteriorated over the time period. This can be observed in Figure 9, which shows a decrease in capacitance relative to the storage time period [4]. The insulating properties of the cell-membrane decrease with the aging period, due to oxidation of phospholipid membrane layers, which makes it permeable [11]. Moreover, the capacitance of membrane was decreases rapidly, about a 30% change in the early aging (0D–7D) period, which shows the significant structural changes in that period.

In contrast, the change in capacitance for the rest of period was not more than 15%, which indicates that cellular membrane became porous [4]. The first observation (0D) was made at 6h postmortem time, and beyond that there were minimal changes in structure once it achieved the ultimate pH level. The obtained data is fitted using a polynomial model, which is given as:

(11) $$C_{1}=5.36377-0.40039\times \text{Aging}+0.0242434\times {\text{Aging}}^{2}-0.000473023\times {\text{Aging}}^{3}\left(\text{pF}\right)$$

**Figure 10:**
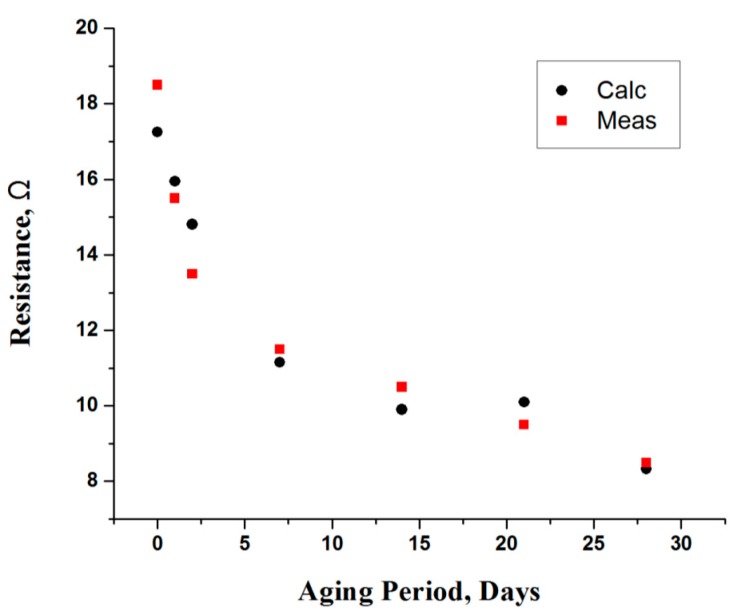
Resistance (R_1_) of an intracellular fluid (ICF) as a function of aging period.

As shown in Figure 9, this expression provides a good approximation of the values found in the measured data, where the overall discrepancy is minimal. Further, as depicted in Figure 10, the resistance of an intercellular fluid (R_1_) decreases during the storage period. This behavior can be explained as reducing the water binding ability of muscle over the storage period. The myofibrillar shrinkage (between thick and thin filaments) reduces the space for intracellular water. Since these myofibrils account for the major portion (about 2/3rd) of muscle water, upon their release and flow between the myofibrils, the conductivity increases. Thus, resistivity decreases with fluid movement as time progresses; however, significant change (42%) is observed in first week of the aging period. The measured data is then used to obtain an empirical model, which is given as:

(12) $${\text{R}}_{1}=17.2538-1.38201\times \text{Aging}+0.0844434\times {\text{Aging}}^{2}-0.00165975\times {\text{Aging}}^{3}\left(\Omega \right)$$

The calculated values of R_1_, which are obtained from expression Equation (12) are in good agreement with the measured data, where the variation in calculated values is negligible (Figure 10).

**Figure 11:**
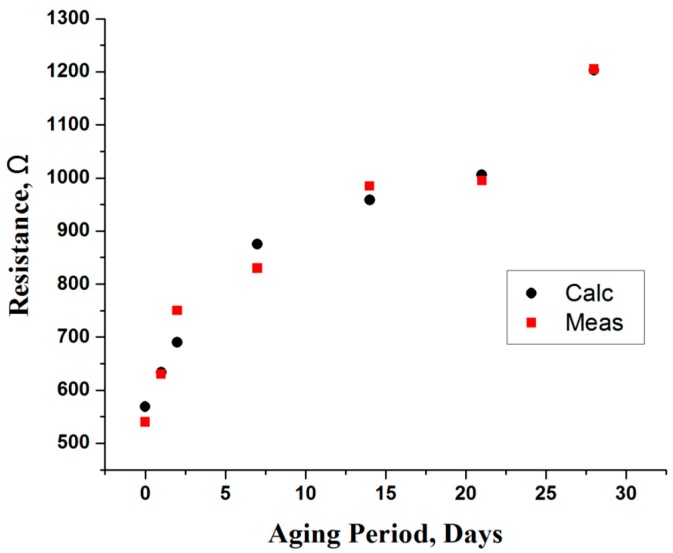
Resistance (R_2_) of an extracellular fluid (ECF) as a function of aging period.

Similarly, the behavior of an extracellular fluid is modeled as resistance (R_2_), where its value progressively increases (Figure 11) during the storage period. The relation of this resistance seems to be related with the drip-loss (comparing with weight-loss in Figure 8). Since, the amount of drip-loss is rapidly increased in the first week of storage, the resistance steadily increases (about 53%), due to a decrease in conductivity. In this period, the loss of water fluid is at the maximum; however, as time progresses the loss tends to decrease, thus changing the resistivity marginally. The empirical model based on polynomials for the extracellular resistance (R_2_) is given by:

(13) $${\text{R}}_{2}=568.811+68.7006\times \text{Aging}-4.19321\times {\text{Aging}}^{2}+0.0910305\times {\text{Aging}}^{3}\left(\Omega \right)$$

As depicted in Figure 11, the calculated values for the extracellular resistance (R_2_) using Equation (13) are in good agreement with measured values, where the overall difference is minimal. Finally, it is deduced that the obtained empirical models for the C_1_, R_1_ and R_2_ can be used to define the relationship between aging and electrical parameters for the aging period (0D–28D) with other considered parameters.

## 5. Conclusions

The effects of aging on broiler meat were studied over a four-week period in which significant changes were observed in resonance frequency and return loss. These changes are even affirmed with weight loss measurements. The anisotropy of muscles was also analyzed, but no significant changes were observed using the proposed resonator. Data from electrical measurements was then evaluated with the Fricke model, which provides a good fit for electric circuit components in biological tissue. Based on the measurement data, empirical models for the circuit parameters were then developed. The measured and calculated values were found to be in good agreement. The results obtained from this research will act as a step towards the development of an effective and reliable microwave sensing device which can be used in meat quality evaluation. Also, the established relationship of equivalent circuit parameters with the physical properties is useful to define electrical characteristics. This will eventually provide an in-depth understanding of dielectric properties associated with the aging period.
